# Pretreatment with nimodipine reduces incidence of POCD by decreasing calcineurin mediated hippocampal neuroapoptosis in aged rats

**DOI:** 10.1186/s12871-018-0501-0

**Published:** 2018-04-16

**Authors:** Qi Zhang, Yanan Li, Yongjuan Bao, Chunping Yin, Xi Xin, Yangyang Guo, Fang Gao, Shuping Huo, Xiuli Wang, Qiujun Wang

**Affiliations:** 1 Department of Anesthesiology, the Third Hospital of Hebei Medical University, No. 139, Ziqiang Road, Shijiazhuang City, 050051 Hebei China; 2 Editorial Department of Chinese Journal of Anesthesiology, Hebei Provincial Institute of Medical Science Information, No. 050071, Western Heping Road, Shijiazhuang City, 050071 Hebei China

**Keywords:** Nimodipine, Calcineurin, Aged, POCD

## Abstract

**Background:**

Calcineurin (CaN) having a high expression in hippocampal neurons is closely related to apoptosis. Pretreatment with nimodipine can lower the apoptosis rate of hippocampal neuron to reduce the incidence of postoperative cognitive dysfunction (POCD). However, the relationship between cerebral protective effect of pretreatment with nimodipine and CaN is controversial in the literature. The aim of this study is to evaluate the relationship between neuroprotective effect of nimodipine and CaN on POCD in aged rats.

**Methods:**

Ninety-six 18-month-old male Sprague-Dawley rats were randomly assigned into 4 groups (*n* = 24 each): control group (Group C), nimodipine group (Group N), surgery group (Group S) and nimodipine + surgery group (Group N + S). In Group N and Group N + S, nimodipine 1 mg/kg was intraperitoneally injected, while the equal volume of normal saline was given instead in Group S. 30 min later, Group N and Group C inhaled pure oxygen for 2 h, and Group S and N + S inhaled 3% sevoflurane for 2 h when exploratory laparotomy was performed. Morris water maze test was performed on 1 day before operation and 1, 3 and 7 days after operation. After the end of Morris water maze test at 1 day before operation and 1 and 7 days after operation, 8 rats were sacrificed, brains were removed and hippocampal tissues were obtained for detection of apoptosis in hippocampal neurons, cytoplasmic calcium ([Ca^2+^]_i_), and hippocampal CaN and caspase-3 expression.

**Results:**

Compared with the 1st day before operation, the escape latency, apoptosis rate, [Ca^2+^]_i_, expression of CaN and caspase-3 increased significantly, but the frequency of crossing the original platform decreased dramatically in Group S and N + S(*P*<0.05). In addition, the escape latency, apoptosis rate, [Ca^2+^]_i_, and expression of CaN and caspase-3 decreased markedly, but the frequency of crossing the original platform increased significantly in Group N + S as compared with Group S (*P*<0.05).

**Conclusions:**

Pretreatment with nimodipine reduces the incidence of POCD by decreasing CaN mediated hippocampal neuroapoptosis in aged rats.

## Background

Postoperative cognitive dysfunction (POCD), a major clinical issue in geriatric surgical patients, with the characteristics of insanity, anxiety, personality changes, memory impairing and so on is one of most common complications of central nervous system in the elderly (≥65 years) [1, 2]. Its incidence varies from 20 to 79% in cardiac surgery and 4.1 to 22.3% in non-cardiac surgery [3, 4]. In recent years, considerable researches have suggested that reactive oxygen species (ROS) [5], hippocampal neuroapoptosis [6], and neuroinflammation [7] are closely related to the pathogenesis of POCD.

The hippocampus is an important component of limbic system which is associated with the regulation of learning, memory, emotion and behaviors [8]. The inflammation reaction, oxidative stress and apoptosis in hippocampal neurons would cause the occurrence of POCD [9]. Sevoflurane is the inhalational anesthetics commonly used in clinical practice with the characteristics of working fast, waking up fast and easy to adjust anesthesia depth and widely used in the elderly. Previous studies have revealed that sevofluane could induce apoptosis in hippocampal neuron and caused cognitive dysfunction persisting for several weeks in aged rats [10, 11].

Calcineurin (CaN) is a calcium/calmodulin-dependent serine/threonine protein kinase, which is sensitive to intracellular calcium ([Ca^2+^]_i_) level change. It is highly expressed in the nervous system, mainly distributed in the hippocampus, caudate nucleus and putamen, secondly distributed in cerebellum and neocorte. A large number of studies have confirmed that CaN is closely related to phosphorylation of Tau protein, synaptic plasticity and neuronal apoptosis [12–14]. Nimodipine which has the special blocking effect on the nerve cell and the brain microvascular endothelial cell is a calcium antagonist of the 1,4-dihydropyridine family [15]. It is highly lipophilic, crosses the blood-brain barrier, and reaches brain and cerebrospinal fluid [16]. Nimodipine may selectively exert a cytoprotective influence by blocking the L-type-calcium channel and reducing calcium influx into nerve cell [17]. Professor Haile M showed that nimodipine could prevent transient cognitive dysfunction after moderate hypoxia in adult mice [18] and improve the disruption of spatial cognition induced by cerebral ischemia in rats [19].

Our previous studies showed pretreatment with nimodipine could prevent POCD by reducing the apoptosis rate of hippocampal neuron in aged rats [20], but whether its mechanism is associated with CaN is still unknown. Therefore, this study aimed to evaluate the relationship between neuroprotective effects of nimodipine pretreatment and CaN.

## Methods

### Animals and grouping

Ninety-six 18-month-old male Sprague-Dawley rats weighing 500–550 g were purchased from the Beijing Weitong Lihua Experimental Animal Technology Co. Ltd. and were randomly assigned into 4 groups (*n* = 24 each): control group (Group C), nimodipine group (Group N), surgery group (Group S) and nimodipine + surgery group (Group N + S). In Group N and Group N + S, nimodipine (Bayer Schering Pharma AG, Germany) 1 mg/kg was intraperitoneally injected, and the equal volume of normal saline was given instead in Group S. 30 min later, Group N and Group C inhaled pure oxygen for 2 h, and Group S and Group N + S inhaled 3% sevoflurane for 2 h when surgery was performed.

### Exploratory laparotomy

Exploratory laparotomy was performed at 30 min after pretreatment. Briefly, after disinfection of the abdomen, a vertical incision of about 3 cm was made on abdominal medial line. Then intestinal, liver, spleen, kidney and other organs were detected in turn. Once no bleeding was confirmed, sterile sutures were used to close the wound, and animals were placed at 37 °C until recovery of consciousness.

### Morris water maze test

Morris water maze test was performed on 1 day before operation and 1, 3 and 7 days after operation to test spatial learning and memory. A circular black and transparent pool (180 cm diameter × 50 cm high and 32 cm deep) was filled with water (24–26 °C). The water was made opaque by the addiction of black nontoxic ink and divided into 4 quadrants equally named I, II, III and IV, and a circular platform (12 cm diameter × 30 cm high) was placed within the pool, submerged 2 cm below the water surface in the middle of quadrant IV. The reference substance around the pool was unchanged and the room lights maintained constant. Rats received a 4-day training before test. In each training day, rats were placed in the pool facing the wall at different four starting locations, the rats failed to find the platform within 120 s would be guided to it and allowed to stay on the platform for 15 s. After each training day, pool was cleaned to eliminate the sense of smell prompt. The training consisted of four sessions per day. On the 5th day and 1, 3 and 7 days after operation, 8 rats of each group were placed in the pool, and the escape latency (from the rat was dropped into water to the rat board the platform) was recorded. Then the platform was removed and frequency of rat swimming across the original location of platform within 60 s was recorded. Morris water maze test was conducted using JLBehv-MWM Morris water maze system (Shanghai Ji’liang Software Technology Co., Ltd).

### Detection of hippocampal neuroapoptosis

On 1 day before operation and 1 and 7 days after operation, 8 rats of each group were sacrificed by intraperitoneal injection of 7% chloral hydrate 2 ml/ kg. The brains of rats were lavaged by cold 0.9% saline isolated from the aorta and hippocampal tissues were quickly separated from brain on the ice. The hippocampal tissues 1 g was filtered through a 200-mesh nylon net and the cell suspensions were centrifuged at 500 rpm at 4 °C for 5 min. The supernatant was removed, and 500 μl of binding buffer was added to prepare single cell suspension (1 × 10^6^/L). Then 5 μl Annexin Vand 10 μl (propidium iodide) PI were added and incubation was done for 5 min in dark. Flow cytometry was performed to detect the apoptotic cells (Beckman Coulter) and the apoptosis rate was calculated.

### Detection of intracellular calcium

Hippocampal tissues were obtained from 8 rats of each group at 1 day before operation and 1 and 7 days after operation to prepare cell suspension by centrifugation for 5 min at 1000 r/min (radius 10 cm) and were resuspended in 3 ml DMEM culture medium to prepare single-cell suspension 1 × 10^5^~ 5 × 10^5^ cells/ml. Then 5 μmol/L Fura-3/AM was added into single-cell suspension and the mixture was incubated at 37 °C for 30 min with shaking. After washing in DMEM twice, cells were resuspended at 37 °C for 15 min. Flow cytometry was performed to detect the fluorescence intensity at excitation wavelength of 480 nm and emission wavelength of 525 nm. Fluorescence index was used to reflect the concentration of [Ca^2+^]_i_ in the cytoplasm.

### Western blot analysis

Hippocampal cells and lyses buffer were homogenized and centrifuged at 12000×g for 10 min at 4 °C, and the protein concentration in the supernatant was determined by BCA assay. The supernatant was decanted and stored at − 70 °C until analysis. Protein concentrations were measured using the Coomassie brilliant blue. 30 μg proteins from the whole cell lysates was separated by sodium dodecyl sulfate-polyacrylamide gel electrophoresis and transferred to a nitrocellulose membrane using Towbin transfer buffer. Blots were blocked overnight with 5% (*w*/*v*) nonfat dry milk in Tris-buffered saline (TBS) and probed a primary antibody for CaN (1:1000, Abcam, Cambridge, UK), caspase-3 (1:500, Abcam, Cambridge, UK) and β-actin (1:10000, Santa Cruz Biotechnology, Inc., Santa Cruz, CA) incubation at 4°Covernight. At 30 min after rewarming on the second day, blots were washed 3 times in TBS and incubated with a goat anti-rabbit horseradish peroxidase secondary antibody (1:10000, Beijing Chinese fir jinqiao biological engineering co., LTD) in dark for 60 min. The blots were developed via enhanced chemiluminescence and were digitally scanned (GS-700, Bio-Rad, USA). To quantitatively assess changes in estimated proteins, the appropriate bands were analyzed by densitometry and quantified by computer analysis.

### Statistical analysis

Statistical analysis was performed with SPSS version 21.0 (SPSS, Inc., Chicago, IL, USA). Quantitative data are expressed as mean ± standard deviation ($\overline{x}$ ± SD). Comparisons between two groups were performed with *t*-test; comparisons among different groups were done with one way analysis of variance (ANOVA). *P*<0.05 was considered statistically significant.

## Results

### Morris water maze test

**Fig. 1:**
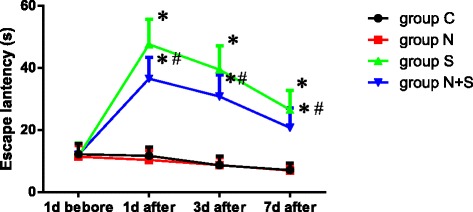
Comparison of escape latency among four groups. ^*^P<0.05 VS 1 d before operation; ^#^*P*<0.05 VS Group S

**Fig. 2:**
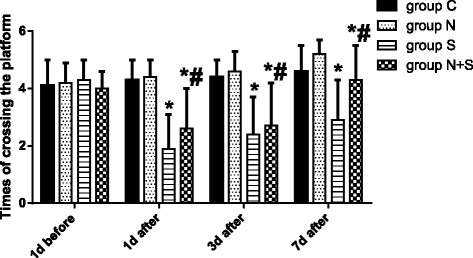
Comparison of the escape latency among 4 groups. ^*^P<0.05 VS 1 d before operation; ^#^*P*<0.05 VS Group S

**Fig. 3:**
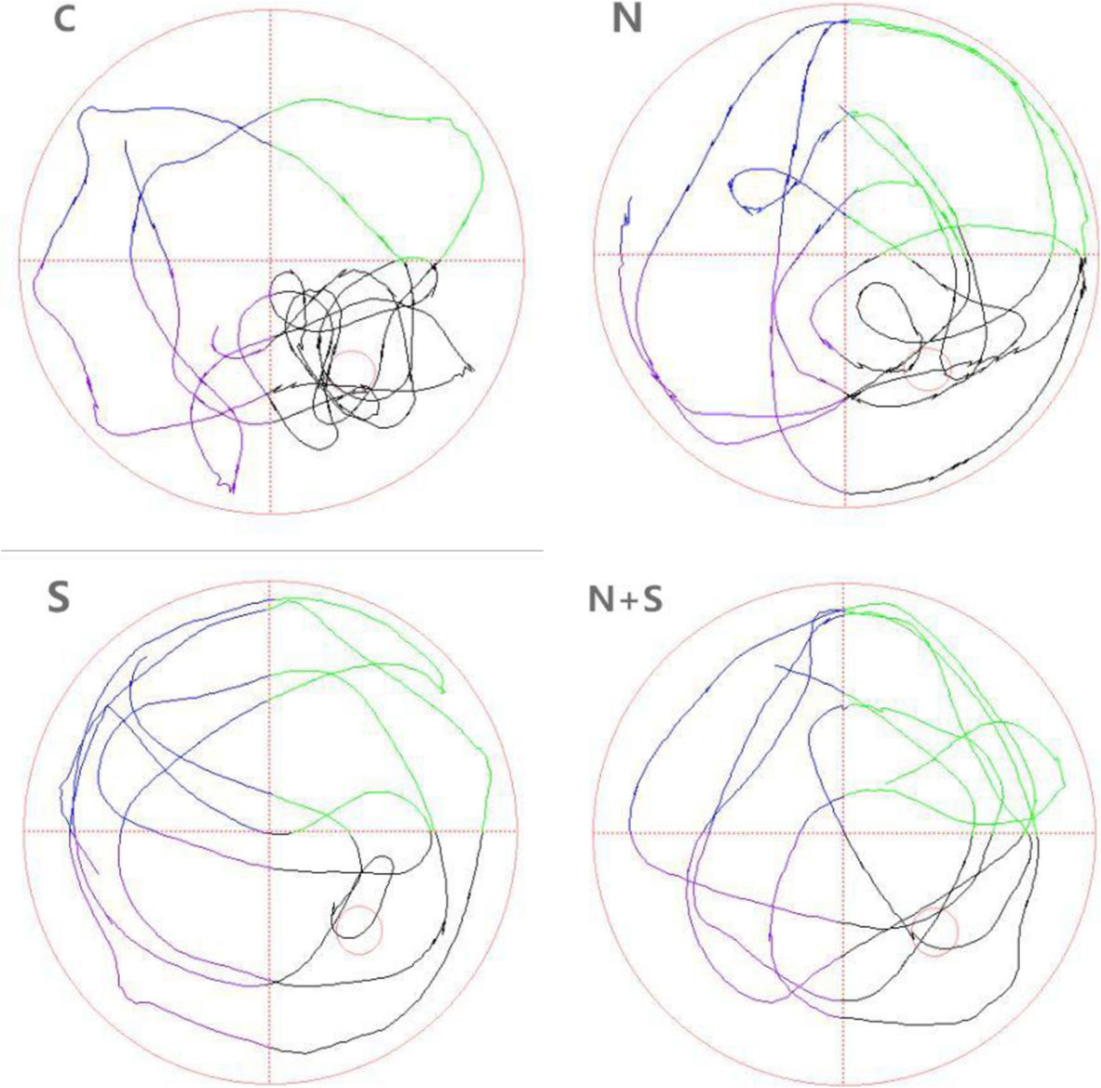
Swimming trajectory of rats at 1 day after operation in different groups. *C* control group, *N* nimodipine group, *S* surgery group, *N + S* nimodipine + surgery group

There were no significant differences in the escape latency and times of crossing the platform among four groups on 1 day before operation (*P* = 0.871, F = 0.135). As shown in Fig. 1 and Fig. 2, compared with 1st day before operation, the escape latency was prolonged, and times of crossing the platform decreased at 1, 3 and 7 days after operation in Group S and Group N + S (Group S: 1 day: *P* = 0.000, *t* = 7.234, 3 day: *P* = 0.005, *t* = 4.031, 7 day: *P* = 0.015, *t* = 3.203; Group N + S: 1 day: *P* = 0.001, *t* = 5.434, 3 day: *P* = 0.011, *t* = 3.487, 7 day: *P* = 0.021, *t* = 2.978). Compared with Group S, the escape latency was decreased, and times of crossing the platform increased at 1, 3 and 7 days after operation in Group N + S (1 day: *P* = 0.013, *t* = 3.297, 3 day: *P* = 0.023, *t* = 2.815, 7 day: 0.038, *t* = 2.581). And the swimming trajectory was shown in Fig. 3.

### Detection of hippocampal neuroapoptosis and [Ca^2+^]_i_

As shown in Table 1., the apoptosis rate and [Ca^2+^]_i_ were comparable before operation among groups(*P* = 0.975, F = 0.073). The apoptosis rate and [Ca^2+^]_i_ increased in Group S at 1 and 7 days after operation and in Group N + S at 1 day after operation (Group S: 1 day: *P* = 0.000, *t* = 6.579,7 day: *P* = 0.020, *t* = 2.996; Group N + S: 1 day: *P* = 0.031, *t* = 2.784). Compared with Group S, the apoptosis rate and [Ca^2+^]_i_ decreased in Group N + S at 1 and 7 day after operation (1 day: P = 0.023, *t* = 2.971, 7 day: 0.042, *t* = 2.560).

**Table 1:** Apoptosis rate and [Ca^2+^]_i_ in hippocampal neurons at different time points in different groups (*n* = 8 each, $\overline{x}$**±***s*)

Group	Apoptosis rate (%)			[Ca^2+^]_i_ (nmol/L)		
	1 d before	1 d after	7 d after	1 d before	1 d after	7 d after
Group C	3.9 ± 1.1	4.1 ± 1.1	4.2 ± 1.2	4.5 ± 1.2	4.6 ± 1.3	4.7 ± 1.6
Group N	4.2 ± 2.2	4.1 ± 0.9	3.8 ± 1.3	4.6 ± 1.2	4.7 ± 2.4	4.5 ± 2.1
Group S	4.4 ± 1.4	20.8 ± 4.4^a^	9.5 ± 2.9^a^	4.9 ± 1.3	21.3 ± 5.7^a^	10.6 ± 3.9^a^
Group N + S	4.1 ± 0.9	11.4 ± 3.5^ab^	5.1 ± 2.1^b^	4.3 ± 0.9	12.1 ± 4.6^ab^	5.5 ± 3.6^b^

^a^*P*<0.05 VS 1 d before operation; ^b^*P*<0.05 VS Group S

### Western blot analysis

**Fig. 4:**
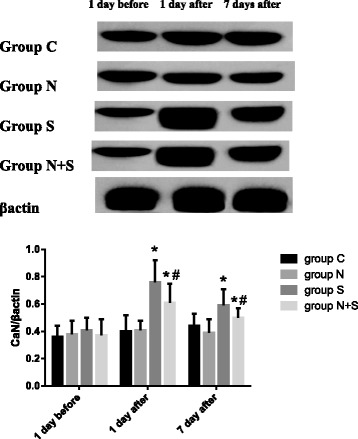
The expression of CaN at different time points. ^*^P<0.05 VS 1 d before operation; ^#^*P*<0.05 VS Group S

**Fig. 5:**
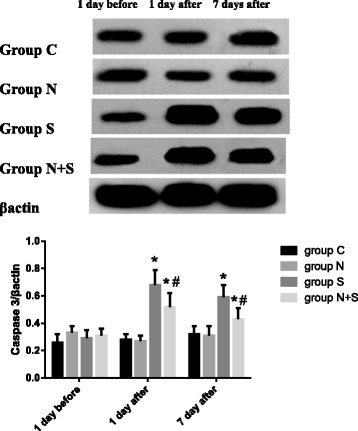
The expression of Caspase-3 at different time points. ^*^P<0.05 VS 1 d before operation; ^#^*P*<0.05 VS Group S

As shown in Fig. 4 and Fig. 5, compared with 1 day before operation, no significant changes were found in the expression of CaN and caspase-3 in Group N and C. The expression of CaN and caspase-3 increased in Group S at 1 and 7 days after operation and in Group N + S at 1 day after operation (CaN: Group S: 1 day: *P* = 0.000, *t* = 5.873, 7 day: *P* = 0.010, *t* = 3.002; Group N + S: 1 day: *P* = 0.002, *t* = 4.790; caspase-3: Group S: 1 day: *P* = 0.000, *t* = 5.507, 7 day: *P* = 0.035, *t* = 3.143; Group N + S: 1 day: *P* = 0.017, *t* = 3.148). Compared with Group S, the expression of CaN and caspase-3 decreased at 1and 7 day after operation (CaN: 1 day: *P* = 0.014, *t* = 3.299, 7 day: *P* = 0.022, *t* = 2.984; caspase-3: 1 day: *P* = 0.011, *t* = 3.449, 7 day: *P* = 0.037, *t* = 2.639).

## Discussion

Our previous study have shown that pretreatment of nimodipine could inhibit calcium overload-induced apoptosis in hippocampal neurons and improve the postoperative cognition of aged rats [20]. This study was undertaken to investigate the mechanism of protective function of nimodipine. Our results further showed that pretreatment with nimodipine could reduce incidence of POCD by decreasing CaN mediated hippocampal neuroapoptosis in aged rats.

Professor P. Vlisides have confirmed that inhalational anesthetics such as sevoflurane or isoflurane have an effect of increasing cytosolic calcium concentration and causing significant neurotoxic effects in hippocampal neurons [21]. Morris water maze test which contains place navigation test and spatial probe test is a classical method to evaluate the spatial cognitive function of rodents [22]. Place navigation test reflects the ability of learning and memory in animal and spatial probe test reflects animal spatial association ability and response memory inquiry ability. Therefore, our study choose Morris water maze test to evaluate the cognition of aged rats.

Nimodipine is a lipophilic calcium antagonist that can easily pass through the blood brain barrier and reach a high concentration in cerebrospinal fluid. Studies have shown that nimodipine could increase cerebral perfusion pressures [23] and improve cognition by reducing neuronal damage [18]. Nimodipine can dilate the cerebral vessels specifically and have almost no effect on peripheral vessels. In our previous study, we found that pretreatment of nimodipine on dose of 1 mg/kg does not cause hypotension and the reduction of cerebral perfusion in rats [20]. As a major intracellular messenger, calcium is involved in the regulation of physiological activities in many cells and tissues, including muscle contraction, metabolism, secretion, and cell division [24]. In the resting state, the [Ca^2+^]_i_ remained at a very low level, about 10^− 7^ mol /L. When stimulation occurs, cells will increase the intracellular concentration of calcium by a variety of ways. Previous studies indicated that [Ca^2+^]_i_ is closely related to neuronal apoptosis [25]. In the study of Xie, results showed prolonged exposures to inhalational anesthetics such as sevoflurane may induce cell damage by apoptosis through direct cytotoxic effects [26]. In aged rats, findings confirmed that sevoflurane-induced neuroapoptosis was mediated by calcium releasing from endoplasmic reticulum to cytoplasm of neurons.

Apoptosis is a kind of active programmed cell death that occurs in the process of the development of cells in the body or under the action of some factors. Direct activation of the Ca^2+^ dependent protease, CaN, appears to represent the target for Ca^2+^ action in apoptosis. CaN-mediated enzyme reaction is a feature of some animal models of neurodegenerative disease, for example, Alzheimer’s disease [27] and cerebral ischemia-reperfusion injury [28]. Caspase-3 which is mainly involved in the process of apoptosis is the main executor of apoptosis. Under normal circumstances, caspase-3 exists in the inactive zymogen. When apoptosis signal appears, caspase-3 transforms from inactive zymogen to active enzymes. Activated caspase-3 could lyse the cell membrane, anti apoptotic protein and prevent the repair of damaged DNA. In the neuronal apoptosis pathway, the calcium-activated CaN activates caspase-3, which ultimately leads to neuronal apoptosis. Using the surface binding of Annexin-V and PI which is one of the commonly used techniques to detect apoptosis, results showed that apoptosis rate in the hippocampus was significantly increased in Group S and Group N + S as compared with Group C. However, apoptosis rate in Group N + S was significantly decreased than that of Group S. It suggested that pretreatment with nimodipine was able to prevent sevoflurane-induced POCD in aged rats receiving surgery via down-regulating the expression of CaN.

## Conclusion

In conclusion, our results indicate that pretreatment with nimodipine may exert neuroprotective effects in aged rats receiving exploratory laparotomy requiring inhalation of sevoflurane. And this protection is, at least partially, related to reduction of CaN mediated hippocampal neuroapoptosis in aged rats.

## Abbreviations

ANOVA: Analysis of variance
CaN: Calcineurin
POCD: Postoperative cognitive dysfunction
ROS: Reactive oxygen species
TBS: Tris-buffered saline
